# A method to inform team sport training activity duration with change point analysis

**DOI:** 10.1371/journal.pone.0265848

**Published:** 2022-03-21

**Authors:** Ben Teune, Carl Woods, Alice Sweeting, Mathew Inness, Sam Robertson

**Affiliations:** 1 Institute for Health and Sport (iHeS), Victoria University, Melbourne, Australia; 2 Western Bulldogs, Melbourne, Australia; Universiti Sains Malaysia, MALAYSIA

## Abstract

Duration is a key component in the design of training activities in sport which aim to enhance athlete skills and physical qualities. Training duration is often a balance between reaching skill development and physiological targets set by practitioners. This study aimed to exemplify change point time-series analyses to inform training activity duration in Australian Football. Five features of player behaviour were included in the analyses: disposal frequency, efficiency, pressure, possession time and player movement velocity. Results of the analyses identified moments of change which may be used to inform minimum or maximum activity durations, depending on a practitioner’s objectives. In the first approach, a univariate analysis determined change points specific to each feature, allowing practitioners to evaluate activities according to a single metric. In contrast, a multivariate analysis considered interactions between features and identified a single change point, reflecting the moment of overall change during activities. Six iterations of a training activity were also evaluated resulting in common change point locations, between 196 and 252 seconds, which indicated alterations to player behaviour between this time period in the training activities conduction. Comparisons of feature segments before and after change points revealed the extent to which player behaviour changed and can guide such duration decisions. These methods can be used to evaluate athlete behaviour and inform training activity durations.

## Introduction

Sport practitioners often use games-based training activities, or drills, to facilitate the development of physiological capacities and skill qualities of team sport athletes [1, 2]. A key component of the design of such training activities relates to their duration, with practitioners needing to consider the appropriate time for skill learning to occur, while balancing physiological targets needed to improve performance and minimise injury risk [3]. When evaluating training duration, contextualising player behaviour as a function of time provides more detailed insights into how and why certain outcomes have occurred [4]. For example, Australian football (AF) players reduce aggregate physical and technical performance following periods of peak physical intensity in match play [5] or during the second half of match play [6]. In football, second half physical activity is influenced by first half activity levels [7]. Accordingly, such insights allow training to be designed more specifically to player activity levels. Suitable time sensitive data analyses may help inform training duration by providing measures of the fluctuation of player behaviour during training activities, which may indicate a decline in the efficacy of the aims of a particular activity. However, specific techniques to achieve this have not yet been applied to support training prescription.

To inform and evaluate training in team sports, data are typically collected from multiple sources, such as player tracking devices or manual annotation. Commonly, these data are reported using aggregate measures such as distance run, average speed or the volume of skill executions [1, 2]. Such measures have also been compared with aggregate match data to determine the extent to which training activities reflect match demands [1, 8, 9]. However, aggregate measures remain limited in utility as they do not represent the fluctuation of such measures as a function of time. In attempts to alleviate this, player speed has been analysed during matches as subsets of varying time periods such as rolling (between one and ten minutes) time windows [10], five-minute blocks [11], sub-phases of play [12] or player on-field stints [13].

Analyses of measures in a continuous format may yield further detailed insights. The use of continuous measurement is further supported by the framework of the constraints-led approach [14]. This framework conceptualises constraints, such as pressure and time, as boundaries of the performer-environment system which shape the emergence of skilled behaviour. Specifically, constraints emerge and decay over varying time scales, and capturing this change over time is crucial in understanding and contextualising athlete behaviour [15, 16]. Accordingly, a continuous time-series analysis, which evaluates changing contextual information and identifies when meaningful change has occurred, could be beneficial in informing training durations.

Change point detection, also known as time series segmentation, is an analytical method of determining specific locations in a time-series when a meaningful change has occurred. This algorithm can be used to detect single or multiple change points and has been widely applied in areas such as medical monitoring and climate change detection [17]. In sport, change point detection has been applied in AF match play to segment player velocity data to identify potential interchange moments [18]. Recent advances to change point detection can also now perform multivariate analysis [19]. In this approach, multiple sequences of data are combined to form a single time series with multiple observations, which allows for the detection of change points common across multiple time series [19]. Multivariate change point detection may be beneficial in sport where multiple sources of data can be integrated to evaluate a single activity [4, 20]. For example, athlete physical and skilled behaviour could be analysed together to detect moments of change within specific team-sport training activities. This may inform activity duration by objectively identifying when skilled and/or physical behaviour deviates meaningfully from specific training objectives. Thus, this study aimed to apply change point analysis as a method to inform team sport training activity duration, exemplified in AF.

## Methodology

### Participants

Participants were a convenience sample of listed players from a single professional AF club (n = 43; 84 ± 8.2 kg; 187 ± 8.1 cm; 24.5 ± 3.6 y). All players were injury free at the time of participation. Ethics approval was obtained from the Victoria University Human Research Ethics Committee (application number: HRE20-138). Written consent was provided by the club to use de-identified data collected as regular procedure during practice.

### Data collection

Data were collected during the 2021 Australian Football League pre-season. Through consultation with coaching staff and the literature [1, 21], five features of player behaviour were identified to evaluate a training activity (disposal frequency, efficiency, pressure, player possession time and player velocity). Skill event data and player tracking data were collected for each training activity repetition (*n* = 6) as it occurred during regular pre-season training sessions. The training activity was a small sided game with even teams, with each team being required to score at opposing ends of the ground. Each repetition ranged from ten to twelve players per team, depending on player availability, with a field area of approximately 90 x 60 m and a minimum duration of four minutes. For each activity repetition, team selection was quasi-randomised by coaching staff to standardise player positions and skill level. Typical AF rules were governed during the activities by a single coach.

To determine velocity during each training activity repetition, each participant wore a 10 Hz Global Positioning System device (Vector S7, Catapult, Catapult Sports Ltd, Melbourne) placed on their backs between their shoulder blades. Each participant wore the same device during all activities to reduce inter-unit error. Upon completion of the training sessions, tracking data was downloaded for each activity using the associated software package (Openfield, v3.3.0). The tracking data comprised a velocity measurement at each 10 Hz timestamp, for each player and activity, before being subsequently exported for analysis.

All data analysis was completed using the *R* programming language with the *RStudio* software [22] (version 1.3.1093). Velocity data was down sampled to a rate of 1 Hz, by calculating the mean velocity across every 10 fixed samples. This sample rate was used to simplify the merging process with skill event data. To determine the movement velocity during each activity repetition, the average velocity across all players was calculated at each 1 Hz timepoint.

To collect the skill event data, each training activity repetition was filmed with a two-dimensional camera (Canon XA25/Canon XA20) at 25 Hz from a side on or behind the goal perspective. After the training sessions, notational software (Hudl Sportscode, v12.2.44) was used to manually quantify the skill event data. A custom code window was used to record each kick or handball (a “disposal”) according to its type (effective or ineffective) and two constraints on the disposal; possession time (<2 s or >2 s) and physical pressure (pressure or absent). Effectiveness was defined in accordance with Champion Data (Melbourne, Pty Ltd), where a handball or kick <40 m was deemed effective, if the intended target retained ball possession. A kick >40 m was deemed effective if kicked to a 50/50 contest or outnumber to the advantage of the attacking team. Possession time was defined as the time period between a player’s ball possession gain and the moment of ball disposal. Pressure was defined as the physical presence of an opposition player within 3 m of the ball-carrier at the time of ball disposal. Two coders notated effectiveness and three coders notated the constraints (pressure and possession time). To assess the reliability of the notational coding, 168 disposals across three activities—observations not used in analysis—were selected for testing. The Kappa statistic [23] resulted in “almost perfect” inter-rater reliability for each variable (>0.8). Intra-rater reliability testing was completed after 14 days which resulted in Kappa statistics ranging from “substantial” (0.67–0.8) to “almost perfect” across all coders (>0.8). All skill event data was then exported with a time-of-day timestamp rounded to the nearest second.

**Fig 1:**
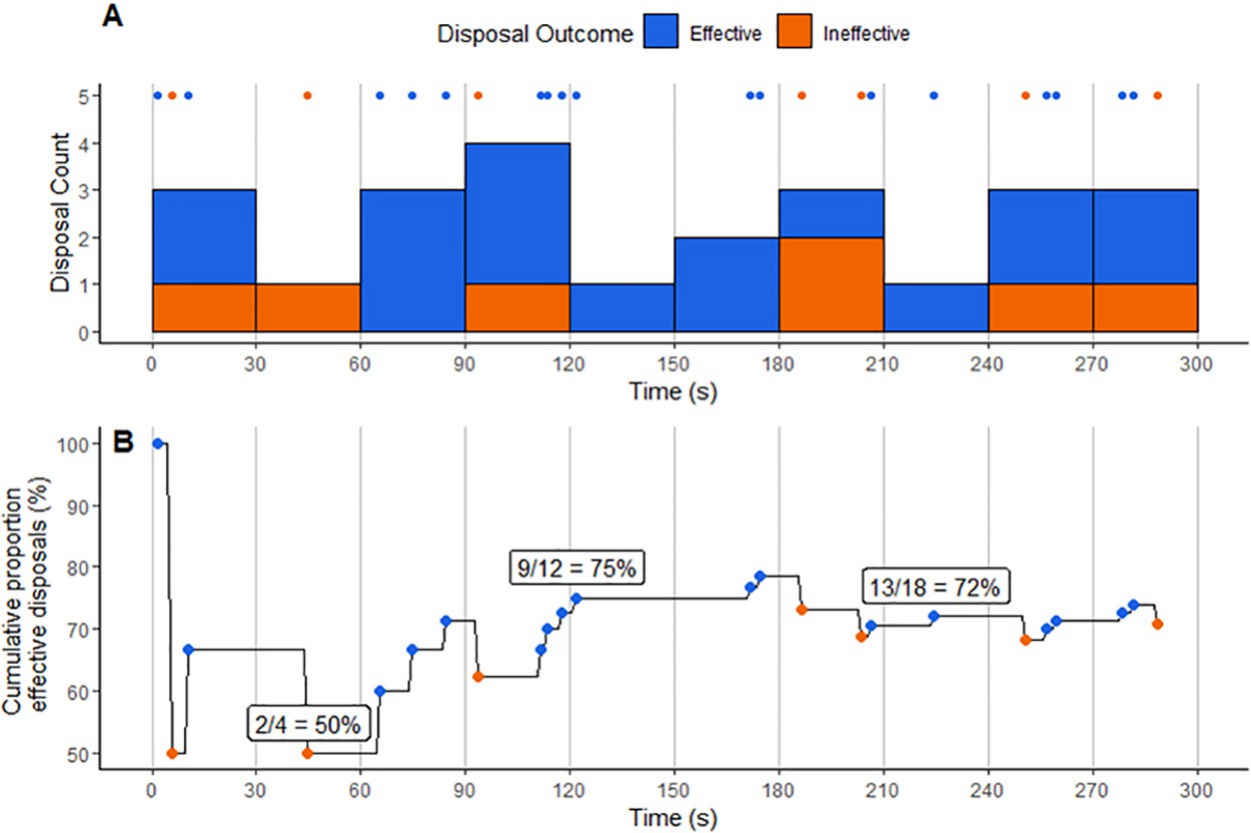
Example from a single activity repetition displaying disposal efficiency represented in 30 s bins (A) and continuously (B). Effective and ineffective disposal events are represented by the points. Three periodic annotations are provided to help describe the sequence calculation in panel B.

For each training activity repetition, the skill event data was joined with the velocity data according to the timestamp. The first and last disposal marked the beginning and end of each activity repetition and was used to determine a relative timestamp in the dataset where each repetition began at zero seconds. To determine disposal frequency as a time series, a rolling sum was applied using a 60 s window. This was achieved using the *rollsum* function from the *zoo* package [24]. A 60 s window was selected as practitioners commonly prescribe activity durations in whole minutes and this function would evaluate a metric analogous to those commonly reported (e.g. metres per minute) in physical training literature [5, 6]. To determine efficiency as a time series, the proportion of cumulative effective disposals to cumulative total disposals was represented as a moving percentage over time. To determine pressure as a time series, the proportion of cumulative pressured disposals to cumulative total disposals was represented as a moving percentage over time. To determine possession time as a time series, the proportion of cumulative disposals with <2 s possession time to cumulative total disposals was represented as a moving percentage over time. This process resulted in four sequences to describe the skilled behaviour during each training activity: disposal frequency (p/min), efficiency (%), pressured disposals (%) and disposals <2 s (%). As an example, efficiency is represented via binning (Fig 1a) and as a continuous series via the above methods (Fig 1b) to contrast the effect of the time series conversion.

### Statistical analysis

To estimate the time point during the activities when properties of the time-series change for each feature, the *cpt_mean* function from the *changepoint* package was used [25]. This function identifies the time point in a sequence where an abrupt change in the sequence mean occurs. The method chosen was AMOC (at most one change) which specifies the algorithm to search for a maximum of one change point in the sequence. This was specified due to the short duration of activities and for feasibility reasons for the end user. The change point algorithm was applied to the sequences of each of the five features for each activity. Each sequence was subsequently segmented according to its change point location.

To determine a single time location common for all features during each activity repetition, a multivariate change point analysis was performed [19]. To achieve this the *mrc* function from the *Changepoint*.*mv* package was used [26]. This function determines common change point locations across multiple sequence inputs of the same length. The features of each training activity were normalised to allow comparison across different measures. The *mrc* function was applied across the normalised feature sequences for each activity. The function parameters were set where the cost was “mean”, specifying the algorithm to search for a change in the sequence means, and the maximum number of change points to search for was set to one. This parameter was chosen to locate a single change point common across all five features of the activity. Each activity was then segmented according to the identified multivariate change point location.

## Results

Descriptive statistics are are presented as means and standard deviations. Across six repetitions of the training activity, the mean duration was 298 ± 17 seconds, disposal frequency was 5.7 ± 1.1 disposals/min, efficiency was 79.5 ± 9.1%, pressure was 40.6 ± 16.3%, possession time was 27.5 ± 19.6% and velocity was 127 ±.7.2 m•min^-1^. The total number of skill involvements and activity duration included in the sample was 185 and 29.2 minutes, respectively.

**Fig 2:**
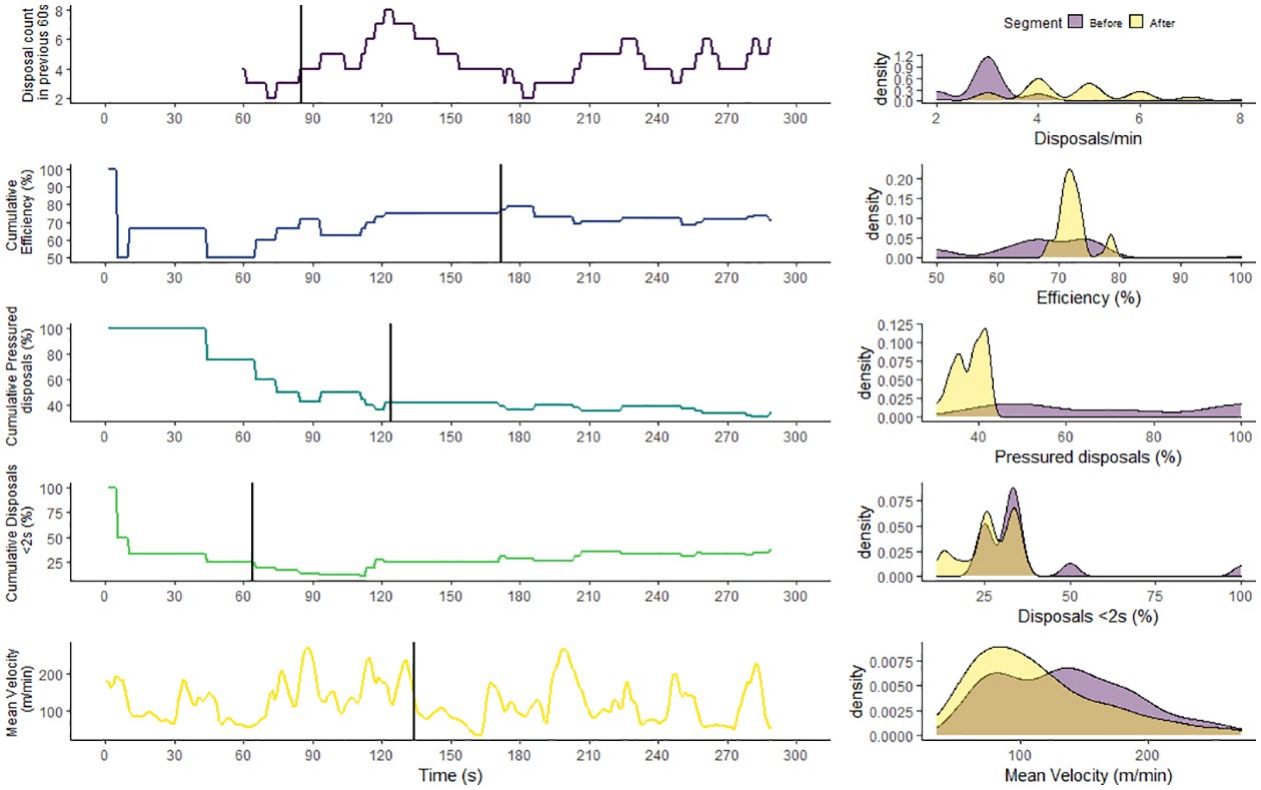
A univariate changepoint analysis of a single training activity. The left-hand column of panels displays the feature and the calculated changepoint location (black vertical line). The right-hand column of panels displays the distribution of the feature in each segment, before and after the changepoint.

**Fig 3:**
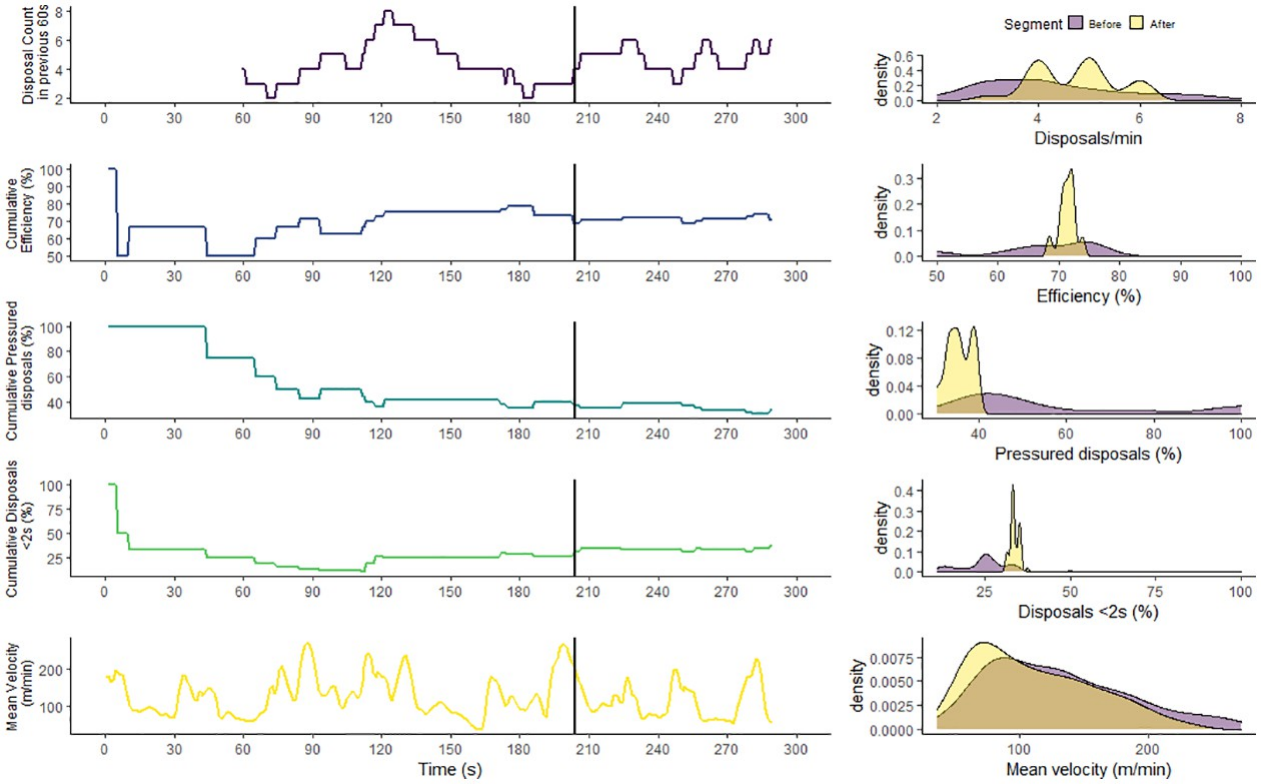
A multivariate changepoint analysis of a single training activity. The left-hand column of panels displays the feature and the calculated changepoint location (black vertical line). The right-hand column of panels displays the distribution of the feature in each segment, before and after the changepoint.

**Fig 4:**
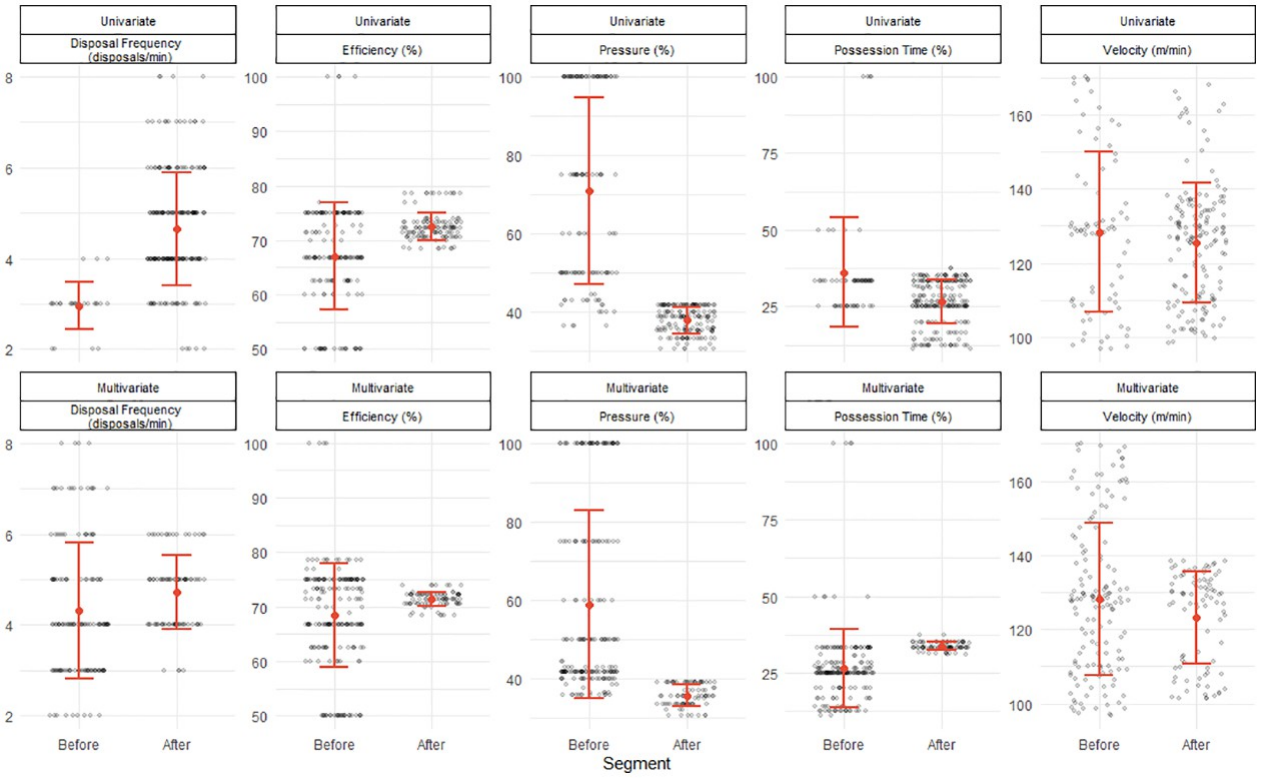
Summary statistics for segmented features according to a univariate and multivariate change point analysis of a single training activity. The orange point and error bars display the mean and one standard deviation of the segment, respectively. The black points each represent one second of the underlying segmented feature.

To demonstrate the univariate and multivariate change point analysis approach, the results for a single activity repetition are reported in Figs 2 and 3, respectively. The left-hand column of panels visualises when the change points occurred and the right-hand column of panels visualises the feature distribution, before and after the change point, to describe the extent of change. The univariate change point analysis of disposal frequency, efficiency, pressure, possession time and velocity resulted in change point located at 85, 172, 124, 64 and 135 s respectively (Fig 2). For each feature the mean and standard deviation of the segments, before and after the changepoint, are reported in Fig 4. The multivariate changepoint approach resulted in a single changepoint for all skill features located at 204 seconds (Fig 3). For each feature the mean and standard deviation of the segments, before and after the changepoint, are reported in Fig 4.

**Fig 5:**
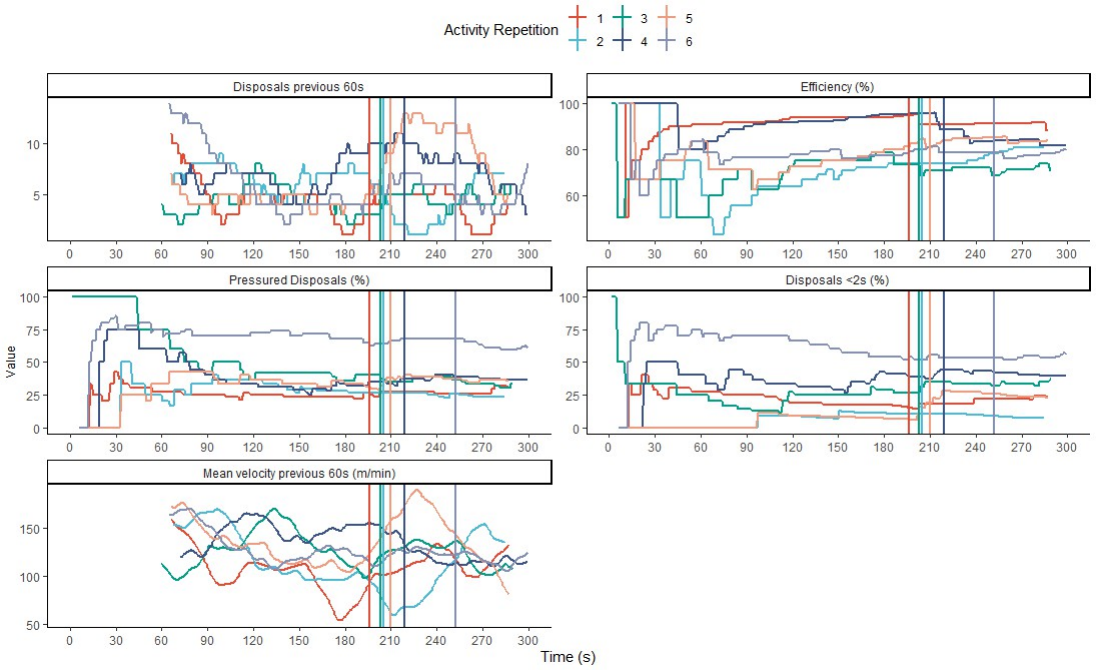
The sequences and multivariate changepoint locations for each feature of six activity repetitions. The feature value through the duration of the activity is displayed with straight vertical lines indicating a change point location. For velocity, the rolling mean over the previous 60 s is displayed to improve its visual interpretability. Feature sequences and changepoint locations are coloured according to activity repetition.

To inform activity duration, the results of the multivariate changepoint analysis on each activity repetition was visualised in Fig 5. Change point locations occurred at 196, 203, 205, 210, 219 and 252 s. Across six repetitions the mean location was 214.2 s with a standard deviation of 20.1 s.

## Discussion

This study presented a univariate and multivariate approach to determining change points during training activities that could be utilised by practitioners to inform training duration. Results demonstrated that the univariate approach was advantageous for providing information specific to each activity feature, which is useful for evaluating training according to a single metric. Comparatively, the multivariate approach is advantageous in analysing the interaction between multiple data sources, providing a simple output for the end user to inform a moment of overall change in the training activity. To guide informed activity duration decisions, visualisations were provided, summarising the univariate change point analysis of six repetitions of the training activity.

In the application of a univariate change point analysis, each of the five features were analysed separately. By resolving the change point location of each feature, a practitioner can identify when each feature meaningfully changed. To know the magnitude of change, the descriptive statistics comparing segments before and after the changepoint are shown in Fig 4. Fig 2 provides an example visualisation which may be useful for practitioners, displaying both when and to what extent each feature has changed during the activity.

According to key ideas of the constraints-led approach, player behaviour is continuously shaped under the interaction of various constraints [14]. The change point analysis used here may, therefore, help practitioners identify periods of behavioural change in a continuous manner. For example, the change point for pressure was identified at 124 seconds, reducing the mean and standard deviation by 33.1% and 20.5%, relatively, after this point (Fig 4). A potential explanation for this observation is the effect of fatigue, which can impact a defending player’s capacity to physically pressure the ball-carrier. Thus, it is possible that defenders may have adapted how they defended—deciding to cover or protect space, rather than chasing the ball-carrier. In this case, the change point could be used to identify how a new behavioural pattern has emerged, which can inform a practitioner’s decisions regarding training design and duration in future iterations of the activity. Indeed, research has measured the aggregate influence of constraints, such as field area [27], game type [28] or playing number [29] on physical and technical behaviour, however this relationship as a function of time presently remains unknown.

Determining a change point for each feature separately does have practical importance, allowing an activity to be evaluated according to a specific metric. For example, if a practitioner is seeking to ensure the efficiency of skills during a practice task does not drop below a certain level, a change point may be useful for noting when a meaningful shift has occurred, thereby allowing them to affect the design of the task. Further, univariate change point analysis has the potential to benefit practitioners with varying responsibilities, such as a conditioning coach and a skills coach. A conditioning coach, for example, can examine the change point for velocity to monitor the physiological demands on the players, while the skills coach can examine the change point for efficiency to monitor the difficulty of the task. This analysis provides a platform for collaboration between coaches to inform the duration of training that provides adequate time to achieve both physiological and skill targets. Importantly, although analysis has occurred separately, each of the features can still be visualised together (Fig 2), further encouraging collaboration between staff when evaluating the activity [20].

The multivariate change point approach analysed the interaction between features and identified one change point common across all features at 204 seconds (Fig 3). In the field, this approach provides a relatively simple output for practitioners, guiding decisions about practice design informed by a “general” or “overall” change point. As shown in Fig 4, the standard deviations of each feature after the change point were reduced. The larger variability of behaviour before the change point may, therefore, indicate exploratory actions of players searching for a stable solution suitable to the constraints of the task [30]. Depending on the practitioner’s objective for the activity, the identified change point may then serve as a potential “cut-off” point for facilitating exploratory player behaviour or a “minimum duration” required to provide adequate time for players to attune to their environment. Thus the change point can serve to analyse acute changes in the learning process to inform training design however, these should be considered within the longer time-scales of the learning process, such as weeks and months [31].

To further support a practitioner’s decision making regarding practice design, six iterations of the same activity were analysed, in a multivariate manner, to evaluate trends in their change points. The visualisation in Fig 5 presents an exemplar technique to communicate information to practitioners on change point locations and feature values during each activity repetition. Five of the six change points appear similar across each repetition (Fig 5). From a practice design perspective, this gives practitioners confidence that during this period there is a change in overall player behaviour. This may serve as a more optimal activity duration to prescribe during future iterations of the activity, reducing time spent in undesired behavioural states. Importantly, variable behavioural states are likely to occur during match play and may reflect a training target for practitioners. The analysis may then aide to increase the efficiency of training sessions by saving valuable training time. In Fig 5, each activity repetition has been colour-coded so practitioners may identify specific results, such as an outlier, if desired. Retrospective inspection of video footage may provide additional information (e.g. weather or player injuries) which may assist in explaining the result.

An important practical aspect of change point analysis is that it accepts various metric representations, such as a rolling mean for disposal frequency and continuous velocity data from player tracking devices. This increases versatility in an environment, such as sport, where multiple data types are common. Specifying the algorithm to search for one change point provides a simple “before” and “after” summary of the data which improves the interpretability for practitioners. Moreover, as the advancement of technology continues in sport, the implementation of an on-line change point analysis could provide further benefits to practitioners, which could be applied to provide real-time feedback during an activity. This could identify the moment a behavioural change occurs to signal the end of an activity rather than relying on a predetermined time. Alternatively, the change point may present a critical moment for practitioner intervention during a practice task. For example, certain constraints could be manipulated to perturb or preserve the efficiency of disposals during a practice task, such as introducing number imbalances to make it easier or harder for a team’s offence. This may be used to disrupt the players transition into a stable state, encouraging further exploration, or can nudge the players towards more optimal stable solutions [32]. Irrespective, this analysis demonstrates how empirical and experiential knowledge of practitioners can be blended, exhibiting a balanced interaction between “man and machine” [33], while still preserving the domain specific expertise of practitioners [34].

Due to the applied nature of this research, there are some limitations. Only four skill features and one physical feature were collected. To increase to the understanding of player behaviour over time, other features, like target pressure, kick distance, high-speed running or sprint frequency could be included. Additionally, parameters for the change point analysis were set to search for one change point. Thus, future work could investigate the impact of multiple change points in analysing behaviour fluctuations during training activities. While a 60 s window was applied to determine disposal frequency, future work could investigate the influence of other rolling windows on the stability of this metric. Further, future work could explore change point analysis between athletes to account for potential individual differences which may exist. This may support the design of player-specific training durations, information which would be of use to conditioning and medical staff when re-integrating players into team training following injury. Finally, increasing the number of activity iterations presented here from six may help alleviate potential confounding factors of results, such as fluctuating weather conditions or teams.

## Conclusion

This study applied a univariate and multivariate change point analysis to inform training duration. The univariate approach provided change points for each feature, information that would be beneficial for practitioners seeking specific guidance on the evaluation of key metrics to inform the duration of training activities. The multivariate approach provided a single time point of general change and may be broadly indicative of players transitioning into different behavioural states. Evaluating multiple repetitions of the same activity is useful for finding trends in behavioural change and can identify critical points during an activity which can guide decisions around activity duration or even constraint manipulation. Given the practicality of the results presented here, practitioners are encouraged to adapt similar analyses to inform their own training designs.

## Supporting information

S1 Data Skill event data and de-identified player velocity data. (ZIP)

## References

[1] Corbett DM, Bartlett JD, O’connor F, Back N, Torres-Ronda L, Robertson S. Development of physical and skill training drill prescription systems for elite Australian Rules football. Sci Med Footb. 2018 Jan 2;2(1):51–7.

[2] Gabbett T, Jenkins D, Abernethy B. Game-Based Training for Improving Skill and Physical Fitness in Team Sport Athletes. Int J Sports Sci Coach. 2009 Jun 1;4(2):273–83.

[3] Vickery W, Nichol A. What actually happens during a practice session? A coach’s perspective on developing and delivering practice. J Sports Sci. 2020 Jul 29;38(24):2765–73. doi: 10.1080/02640414.2020.1799735 32723022

[4] Glazier PS. Towards a Grand Unified Theory of sports performance. Hum Mov Sci. 2017 Dec 1;56:139–56. doi: 10.1016/j.humov.2015.08.001 26725372

[5] Black GM, Gabbett TJ, Naughton GA, McLean BD. The effect of intense exercise periods on physical and technical performance during elite Australian Football match-play: A comparison of experienced and less experienced players. J Sci Med Sport. 2016 Jul 1;19(7):596–602. doi: 10.1016/j.jsams.2015.07.007 26315892

[6] Black GM, Gabbett TJ, Naughton G, Cole MH, Johnston RD, Dawson B. The Influence of Contextual Factors on Running Performance in Female Australian Football Match-Play. J Strength Cond Res. 2019 Sep;33(9):2488–95. doi: 10.1519/JSC.0000000000002142 28704310

[7] Sparks M, Coetzee B, Gabbett JT. Variations in high-intensity running and fatigue during semi-professional soccer matches. Int J Perform Anal Sport. 2016 Apr 1;16(1):122–32.

[8] Browne PR, Woods CT, Sweeting AJ, Robertson S. Applications of a working framework for the measurement of representative learning design in Australian football. PLOS ONE. 2020 Nov 30;15(11):e0242336. doi: 10.1371/journal.pone.0242336 33253204 PMC7703947

[9] Ireland D, Dawson B, Peeling P, Lester L, Heasman J, Rogalski B. Do we train how we play? Investigating skill patterns in Australian football. Sci Med Footb. 2019 Oct 2;3(4):265–74.

[10] Clarke AC, Whitaker M, Sullivan C. Evolving peak period, match movement, and performance demands in elite women’s Australian football. J Sci Med Sport. 2021 Jul 1;24(7):683–8. doi: 10.1016/j.jsams.2021.01.006 33531273

[11] Carling C, Dupont G. Are declines in physical performance associated with a reduction in skill-related performance during professional soccer match-play? J Sports Sci. 2011 Jan 1;29(1):63–71. doi: 10.1080/02640414.2010.521945 21077004

[12] Rennie MJ, Kelly SJ, Bush S, Spurrs RW, Austin DJ, Watsford ML. Phases of match-play in professional Australian Football: Distribution of physical and technical performance. J Sports Sci. 2020 Apr 28;0(0):1–8. doi: 10.1080/02640414.2020.1754726 32342727

[13] Corbett DM, Sweeting AJ, Robertson S. Weak Relationships between Stint Duration, Physical and Skilled Match Performance in Australian Football. Front Physiol [Internet]. 2017 [cited 2021 Feb 25];8. Available from: https://www.frontiersin.org/articles/10.3389/fphys.2017.00820/full?report=reader 29109688 10.3389/fphys.2017.00820 PMC5660114

[14] Davids K, Button C, Bennett SJ. Dynamics of skill acquisition: a constraints-led approach [Internet]. Champaign, Illinois: Human Kinetics; 2008 [cited 2019 Jun 20]. http://www.humankinetics.com/products/showproduct.cfm?isbn=9780736036863

[15] Newell KM. Constraints on the development of coordination. In: Wade M, Whiting H, editors. Motor Development in children: Aspects of coordination and control. Dordrecht, The Netherlands: Martinus Nijhoff; 1986. p. 341–60.

[16] Balagué N, Pol R, Torrents C, Ric A, Hristovski R. On the Relatedness and Nestedness of Constraints. Sports Med—Open. 2019 Feb 11;5(1):6. doi: 10.1186/s40798-019-0178-z 30742241 PMC6370894

[17] Aminikhanghahi S, Cook DJ. A survey of methods for time series change point detection. Knowl Inf Syst. 2017 May 1;51(2):339–67. doi: 10.1007/s10115-016-0987-z 28603327 PMC5464762

[18] Corbett DM, Sweeting AJ, Robertson S. A change point approach to analysing the match activity profiles of team-sport athletes. J Sports Sci. 2019 Jul 18;37(14):1600–8. doi: 10.1080/02640414.2019.1577941 30747582

[19] Bardwell L, Fearnhead P, Eckley IA, Smith S, Spott M. Most Recent Changepoint Detection in Panel Data. Technometrics. 2019 Jan 2;61(1):88–98.

[20] Browne P, Sweeting AJ, Woods CT, Robertson S. Methodological Considerations for Furthering the Understanding of Constraints in Applied Sports. Sports Med—Open. 2021 Apr 1;7(1):22. doi: 10.1186/s40798-021-00313-x 33792790 PMC8017066

[21] Teune B, Woods C, Sweeting A, Inness M, Robertson S. The influence of environmental and task constraint interaction on skilled behaviour in Australian Football. Eur J Sport Sci. 2021 Jul 26;0(ja):1–20. doi: 10.1080/17461391.2021.1958011 34304723

[22] R Core Team. R: A Language and Environment for Statistical Computing [Internet]. Vienna, Austria: R Foundation for Statistical Computing; 2019. https://www.R-project.org/

[23] Landis JR, Koch GG. The Measurement of Observer Agreement for Categorical Data. Biometrics. 1977;33(1):159–74. 843571

[24] Zeileis A, Grothendieck G. zoo: S3 Infrastructure for Regular and Irregular Time Series. J Stat Softw. 2005 May 21;14(1):1–27.

[25] Killick R, Eckley IA. changepoint: An R Package for Changepoint Analysis. J Stat Softw. 2014 Jun 25;58(1):1–19.

[26] Bardwell L, Eckley I, Fearnhead P, Grose D. changepoint.mv: Changepoint Analysis for Multivariate Time Series [Internet]. 2020 [cited 2021 Jul 13]. https://CRAN.R-project.org/package=changepoint.mv

[27] Fleay B, Joyce C, Banyard H, Woods C. Manipulating Field Dimensions During Small-sided Games Impacts the Technical and Physical Profiles of Australian Footballers. J Strength Cond Res. 2018 Jul 1;32(7):2039–44. doi: 10.1519/JSC.0000000000002423 29337834

[28] Nunes NA, Gonçalves B, Davids K, Esteves P, Travassos B. How manipulation of playing area dimensions in ball possession games constrains physical effort and technical actions in under-11, under-15 and under-23 soccer players. Res Sports Med. 2020 May 26;0(0):1–15. doi: 10.1080/15438627.2020.1770760 32452730

[29] Bonney N, Ball K, Berry J, Larkin P. Effects of manipulating player numbers on technical and physical performances participating in an Australian football small-sided game. J Sports Sci. 2020 Jul 1;30(21):2430–6. 10.1080/02640414.2020.1787697 32605432

[30] Davids K, Glazier P, Araújo D, Bartlett R. Movement systems as dynamical systems: the functional role of variability and its implications for sports medicine. Sports Med Auckl NZ. 2003;33(4):245–60. doi: 10.2165/00007256-200333040-00001 12688825

[31] Sullivan MO, Woods CT, Vaughan J, Davids K. Towards a contemporary player learning in development framework for sports practitioners. Int J Sports Sci Coach. 2021 Mar 22;17479541211002336.

[32] Chow JY. Nonlinear Learning Underpinning Pedagogy: Evidence, Challenges, and Implications. Quest. 2013 Oct 1;65(4):469–84.

[33] Robertson PS. Man & machine: Adaptive tools for the contemporary performance analyst. J Sports Sci. 2020 Jun 12;38(18):2118–26. doi: 10.1080/02640414.2020.1774143 32530736

[34] Greenwood D, Davids K, Renshaw I. Experiential knowledge of expert coaches can help identify informational constraints on performance of dynamic interceptive actions. J Sports Sci. 2014 Feb 25;32(4):328–35. doi: 10.1080/02640414.2013.824599 24016400

